# Comparative study of clinical symptoms of chronic rhinosinusitis with nasal polyps patients

**DOI:** 10.1002/iid3.773

**Published:** 2023-02-16

**Authors:** Jingmin Hu, Le Wang, Ruixia Ma

**Affiliations:** ^1^ Department of ENT The First Hospital of Yinchuan Yinchuan China

**Keywords:** chronic rhinosinusitis, eosinophilic granulocyte, nasal polyps, specific IgE

## Abstract

**Aim:**

The aim of this study is to explore the clinical characteristics in chronic rhinosinusitis with nasal polyps (CRSwNP) patients with different serum specific IgE (SIgE) and eosinophilic granulocyte infiltration status.

**Methods:**

This retrospective observational study included patients diagnosed with CRSwNP and underwent functional endoscopic sinus surgery at the Yinchuan First People's Hospital between June 2019 and June 2021. A total of 192 patients with CRSwNP were included (100 males). The patients were 41.7 ± 15.1 years old. The disease course ranged 4.6–18.2 months. The visual analog scale (VAS) score (*p* < .001), endoscopic score (*p* = .014), computerized tomography (CT) score (*p* < .00) and the sino‐nasal outcome Test‐22 (SNOT‐22) score (*p* < .001) were significantly different among patients with SIgE‐positive combined eosinophilic chronic rhinosinusitis (ECRS), patients with SIgE‐positive combined non‐ECRS, patients with SIgE‐negative combined ECRS, and patients with SIgE‐negative combined non‐ECRS.

**Results:**

In pairwise comparison, the VAS score (20.72 ± 2.24 vs. 13.09 ± 1.62, 13.84 ± 1.34, and 12.67 ± 1.20, respectively), endoscopic score (8.09 ± 1.04 vs. 7.06 ± 0.98, 7.69 ± 1.18, and 7.75 ± 1.07, respectively), CT score (13.18 ± 1.66 vs. 8.79 ± 0.88, 11.08 ± 1.12, and 11.08 ± 1.12, respectively), and SNOT‐22 score (27.62 ± 2.31 vs. 12.09 ± 1.83, 14.84 ± 1.84, and 12.97 ± 1.50, respectively) was significantly higher in patients with SIgE‐positive combined ECRS compared to patients with SIgE‐positive combined non‐ECRS, patients with SIgE‐positive combined non‐ECRS (all *p* < .0083). The VAS score, endoscopic score, and CT score might be higher in CRSwNP patients with SIgE‐positive combined ECRS.

**Conclusion:**

In this study, the VAS score, endoscopic score, and CT score were higher in the patients with SIgE‐positive combined ECRS. This study might provide a reference for treatment in patients with CRSwNP.

## Graphical abstract

F I G U R E 1 (*n* = 192) Patients with CRS_w_NP were included in the study, including 100 males and 92 females. Positive SIgE was found in 105 cases. Among the 105 SIgE‐positive patients, 41 had ECRS, and 64 had non‐ECRS CRS_w_NP. Among the 87 SIgE‐negative patients, 33 had ECRS, and 54 had non‐ECRS CRS_w_NP. There were no significant differences (*p*=.290) between the two groups (A). There was no significant difference in age, sex, endoscopic score, CT score, serum eosinophil count between the two groups (*p* > .05), but the VAS score was significantly higher in the SIgE‐positive group compared with the SIgE‐negative group (■*p* < .001) (B).

F I G U R E 2 Four groups were defined according to ECRS and SIgE: SIgE‐positive combined ECRS, SIgE‐positive combined non‐ECRS, SIgE‐negative combined ECRS, and SIgE‐negative combined non‐ECRS. VAS score (*p* < .001), endoscopic score (*p* = .014), computerized tomography (CT) score (*p* < .001) and SNOT‐22 score (*p* < .001) were significantly different among groups. In pairwise comparison, the VAS score, endoscopic score, CT score, and SNOT‐22 score was significantly higher in patients with SIgE‐positive combined ECRS compared to patients with SIgE‐positive combined non‐ECRS, patients with SIgE‐positive combined non‐ECRS (*p* < .0083). And patients with SIgE‐negative combined ECRS have significantly higher CT score compared to patient with SIgE‐positive combined non‐ECRS and SIgE‐negative combined non‐ECRS.

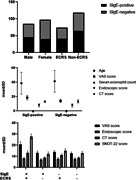

## 1 INTRODUCTION

Chronic rhinosinusitis is a symptomatic inflammation of the nasal passages and paranasal sinuses lasting ≥12 weeks.^1^, ^2^, ^3^ Chronic rhinosinusitis is common, with prevalence estimates ranging from 5% to 15%.^2^, ^4^, ^5^ It is reported as one of the most common reasons for ambulatory care visits.^3^, ^5^ Chronic rhinosinusitis can be associated with sinus outflow obstruction or infection.^4^ Chronic rhinosinusitis is a highly heterogeneous disease with complex etiology and is often accompanied by nasal polyps (CRS_w_NP).^2^, ^4^, ^6^ Indeed, about 20% of cases of chronic sinusitis are reported to have nasal polyps.^5^ The predisposing factors include sinus ostium obstruction, infection, mucosal barrier immune disorder, ciliary dysfunction, and allergic reaction.^1^, ^2^, ^3^ The edematous mucosa forms single or multiple nasal polyps in the nasal cavity and the middle nasal meatus of the sinus.^4^

The impact of allergic reactions on the occurrence and development of CRS_w_NP is still inconclusive.^7^, ^8^ Still, available data support that serum IgE is associated with CRS_w_NP.^9^, ^10^, ^11^ One of the characteristics of the late reaction phase of an allergic reaction is the large number of eosinophilic granulocytes that infiltrate the tissues and act as effector cells.^12^ Eosinophilic chronic rhinosinusitis (ECRS) is a subgroup of CRSwNP and is associated with severe eosinophilic infiltration and Comprehensive treatment based on surgery is required.^13^ Indeed, several studies showed that ECRS has a high recurrence rate after surgery and requires comprehensive treatment.^14^, ^15^

Serum specific IgE (SIgE) detection has been widely used as an objective indicator of allergic reaction and recognized as the golden standard for allergen detection. Previous case‐control studies suggested a possible role of food allergy in CRS_w_NP, with 70% and 81% positivity in the CRS_w_NP groups versus 34% and 11% in the control groups.^16^, ^17^ Still, the association between tissue eosinophilic granulocyte and SIgE in patients with CRS_w_NP remains poorly understood.

Therefore, this retrospective observational study aimed to explore the clinical characteristics in CRSwNP patients with different SIgE and eosinophilic granulocyte infiltration status.

## 2 MATERIALS AND METHODS

### 2.1 Study design and patients

This retrospective observational study included patients diagnosed with CRS_w_NP and underwent functional endoscopic sinus surgery at the ENT & HN Surgery Department of the Yinchuan First People's Hospital between June 2019 and June 2021. This study was approved by the Ethics Committee of the First People's Hospital of Yinchuan City. The requirement for individual consent was waived by the committee because of the retrospective nature of the study.

The diagnostic criteria were in line with the Chinese Guidelines for the Diagnosis and Treatment of Chronic Rhinosinusitis (2018).^18^ Ask each patient in the chronic rhinosinusitis group about their main medical history and complications. The patient's age, sex, course of disease, main symptoms (nasal congestion, purulent discharge, head and face pain, hypoesthesia, etc.), previous relevant surgery history of nasosinusitis and nasal polyps, preoperative history of hormone, anti allergy and other drugs, and whether they are associated with asthma, allergic dermatitis and other allergic reaction diseases were recorded.

The inclusion criteria for the study were (1) >18 years of age, (2) diagnosed with CRS_w_NP, (3) underwent functional endoscopic sinus surgery, and (4) available SIgE data. The exclusion criteria were (1) used systemic glucocorticoids 2 weeks before the surgery, (2) history of nasal or sinus tumors, (3) orally taking antihistamines, (4) fungal sinusitis, or (5) combined with rheumatoid disease.

### 2.2 Data collection **and definition**

The demographic information (including age and sex) and clinical characteristic (including SIgE test results, serum eosinophil count, eosinophilic granulocytes, visual analog scale [VAS] score, computerized tomography [CT] scoeosinophilre, endoscopic score, the sino‐nasal outcome Test‐20 [SNOT‐22] score) were collected.

The SIgE test results before surgery were collected. The semi‐quantitative detection unit of specific allergen IgE was IU/mL, and the grades were classified into: grade 0, normal, <0.35; grade 1, mild, 0.35–0.7; grade 2, moderate, 0.7–3.5; grade 3, severe, >3.5. Grade 0 was considered negative, and grades 1–3 were considered positive.

The subjective symptoms of rhinosinusitis (nasal obstruction, nasal discharge, and swelling pain in the nasal and facial area) of all the patients before surgery were scored using a VAS score. The range from 0 to 10 represented symptoms gradually aggravating.

The eosinophilic granulocytes in the nasal polyp tissue of the enrolled patients after surgery were counted. If the count was ≥10/HPF, it was defined as ECRS, and if the count was <10/HPF, it was defined as non‐ECRS CRS_w_NP.^19^

The Lund‐Mackay CT score^19^ was used to grade the extent of lesions on the coronal CT of the paranasal sinus of the patients before surgery, including the bilateral anterior and posterior sinuses and the ostiomeatal complexes. For the paranasal sinus, 0 represented normal, 1 represented partial shadow, and 2 represented complete shadow. For the ostiomeatal complex, 0 represented no obstruction, and 2 represented obstruction. A maximum of 12 points were scored for each side.

The Lund‐Kennedy score^19^ was used to grade the preoperative nasal endoscopy of the patients as endoscopic score, which was divided into three grades: 0 meant that no nasal polyp was found; 1 meant that the size of the nasal polyp did not exceed the middle nasal concha; 2 meant that the lower end of the nasal polyp exceeded the middle nasal concha. Edema: 0 point, no edema; 1 point, slight edema; 2 points, severe edema. Discharge: 0 point, no rhinorrhea; 1 point, clear and thin nose leakage; 2 points, viscous and purulent rhinorrhea.

SNOT‐20^20^ score was used to score the preoperative questionnaire investigation of the enrolled patients. The Chinese version of SNOT‐22 includes a total of 22 symptoms, involving three aspects: physiological problems, functional limitations and emotional outcomes. The respondents were asked to score 22 symptoms according to their own symptoms of sinusitis. A score of 0 means that they have no problems; 1 point means that the respondent is slightly troubled; 2 points represent moderate distress to the respondents; A score of 3 indicates that the respondents are seriously troubled. The higher the total score of 20 symptoms, the worse the quality of life of the respondents.

### 2.3 Statistical analysis

SPSS 14.0 (SPSS Inc.) was used for statistical analysis. The continuous data was tested for normal distribution by Kolmogorov–Smirnov test and tested for homogeneity of variance by Levene's test. All continuous data confirmed to normal distribution and are expressed as means ± standard deviation and were compared by one‐way analysis of variance test, and Bonferroni's post hoc test was used for pairwise comparisons between groups. Two‐sided *p* < .05 were considered statistically significant.

## 3 RESULTS

A total of 192 patients with CRS_w_NP were included in the study, including 100 males and 92 females. The patients were 18–64 years old, with an average age of 41.7 ± 15.1 years, and the courses of disease ranged from 4.6 to 18.2 months. Positive SIgE was found in 105 cases (54.7%). There was no significant difference in age, sex, endoscopic score, CT score, serum eosinophil count, and ECRS between the two groups (*p* > .05), but the VAS score and SNOT‐22 score was significantly higher in the SIgE‐positive group compared with the SIgE‐negative group (18.5 ± 3.1 vs. 13.9 ± 2.2, *p* < .001) (Table 1).

**Table 1:** Characteristics of the patients.

Characteristics	SIgE‐positive (*n* = 105)	SIgE‐negative (*n* = 87)	*p*
Age, years, mean ± *SD*	38.5 ± 15.1	41.3 ± 15.2	.406
Sex, *n*			.892
Male	45	40	
Female	40	57	
VAS, mean ± *SD*	18.5 ± 3.1	13.9 ± 2.2	<.001
Serum eosinophil count mean ± *SD*	0.21 ± 0.11	0.20 ± 0.17	.6
Endoscopic score, mean ± *SD*	7.8 ± 2.2	7.6 ± 2.2	.743
CT score, mean ± *SD*	12.9 ± 1.2	13.2 ± 2.2	.394
ECRS, *n* (%)			.290
ECRS	41 (39.1)	33 (37.9)	
Non‐ECRS	64 (60.9)	54 (62.1)	

Abbreviations: CT, computerized tomography; ECRS, eosinophilic chronic rhinosinusitis; *SD*, standard deviation; SIgE, serum specific IgE; VAS, visual analog scale.

Among the 105 SIgE‐positive patients, 41 had ECRS, and 64 had non‐ECRS CRS_w_NP. Among the 87 SIgE‐negative patients, 33 had ECRS, and 54 had non‐ECRS CRS_w_NP. There were no significant differences (*p* = .290) between the two groups (Table 1).

Four groups were defined according to ECRS and SIgE: SIgE‐positive combined ECRS, SIgE‐positive combined non‐ECRS, SIgE‐negative combined ECRS, and SIgE‐negative combined non‐ECRS. The VAS score (*p* < .001), endoscopic score (*p* = .014), CT score (*p* < .001) and SNOT‐22 score (*p* < .001) were significantly different among groups. In pairwise comparison, the VAS score (20.72 ± 2.24 vs. 13.09 ± 1.62, 13.84 ± 1.34, and 12.67 ± 1.20, respectively), endoscopic score (8.09 ± 1.04 vs. 7.06 ± 0.98, 7.69 ± 1.18, and 7.75 ± 1.07, respectively), CT score (13.18 ± 1.66 vs. 8.79 ± 0.88, 11.08 ± 1.12, and 11.08 ± 1.12, respectively), and SNOT‐22 score (27.62 ± 2.31 vs. 12.09 ± 1.83, 14.84 ± 1.84, and 12.97 ± 1.50, respectively) was significantly higher in patients with SIgE‐positive combined ECRS compared to patients with SIgE‐positive combined non‐ECRS, patients with SIgE‐positive combined non‐ECRS (all *p* < .0083). And patients with SIgE‐negative combined ECRS have significantly higher CT score compared to patient with SIgE‐positive combined non‐ECRS and SIgE‐negative combined non‐ECRS (Table 2).

**Table 2:** VAS score, endoscopic score, and CT score according to the combinations of SIgE and ECRS.

Characteristics, mean ± SD	SIgE‐positive combined ECRS (*n* = 41)	SIgE‐positive combined non‐ECRS (*n* = 64)	SIgE‐negative combined ECRS (*n* = 33)	SIgE‐negative combined non‐ECRS (*n* = 54)	*p*
VAS score	20.72 ± 2.24	13.09 ± 1.62^a^	13.84 ± 1.34^a^	12.67 ± 1.20^a^	<.001
Endoscopic score	8.09 ± 1.04	7.06 ± 0.98^a^	7.69 ± 1.18^a^	7.75 ± 1.07^a^	.014
CT score	13.18 ± 1.66	8.79 ± 0.88^a^	11.08 ± 1.12^a^,^b^	8.75 ± 0.94^a^	<.001
SNOT‐20 score	27.62 ± 2.31	12.09 ± 1.83^a^	14.84 ± 1.84^a^	12.97 ± 1.50^a^	<.001

*Note*: SNOT‐22 = the sino‐nasal outcome Test‐22.

Abbreviations: CT, computerized tomography; ECRS, eosinophilic chronic rhinosinusitis; *SD*, standard deviation; SIgE, serum specific IgE; VAS, visual analog scale.

a *p* < .05 versus SIgE‐positive ECRS.

^b^ *p* < .05 versus SIgE‐positive non‐ECRS and SIgE‐negative non‐ECRS.

## 4 DISCUSSION

In this study, the VAS score, endoscopic score, and CT score were higher in the patients with SIgE‐positive combined ECRS. This study might provide a reference for treatment in patients with CRS_w_NP.

At present, it is generally believed that the detection of SIgE or laboratory‐specific IgE is safer and more reliable than the skin‐prick test and is the best indicator for the diagnosis of allergy or allergy‐related diseases.^21^ In this study, SIgE was used as an objective indicator as to whether patients with CRS_w_NP had an allergy or not. The subjects were divided into the SIgE‐positive and SIgE‐negative groups. There were no significant differences in the objective scores of Lund‐Mackay CT and Lund‐Kennedy endoscopy between the two groups, but the subjective symptoms based on the VAS were worse in the presence of allergy. These results suggest that when CRS_w_NP patients are combined with allergy, their subjective symptoms such as nasal obstruction, nasal discharge, and swelling pain in the nasal and facial area (which are not assessed by CT and endoscopy) are aggravated even in the absence of significant differences in CT and endoscopic scores. Szucs et al.^22^ also pointed out that the severity of adult CRS_w_NP and the difficulty in treatment are positively related to the infiltration of eosinophilic granulocyte in the tissue but not related to the patient's allergic constitution, which contradicts the present study. On the other hand, Collins et al.^16^ and Pang et al.^17^ showed a higher proportion of CRSwNP patients with allergies than controls, supporting the present study.

Snidvongs et al.^19^ suggested that an increase in the number of eosinophilic granulocytes in the peripheral blood or tissue of CRS_w_NP patients cannot provide a conclusion about the allergy status. It is currently believed that the increased number and degree of infiltration and aggregation of eosinophilic granulocyte is related to the eosinophilic chemokines released by basophilic granulocyte or mastocyte at the site of allergy.^12^ Demoly et al.^23^ found no significant differences in the degree of eosinophilic infiltration between CRS_w_NP patients with allergy and without allergy. Snidvongs et al.^19^ believed that when tissue eosinophilic granulocyte is ≥10/HPF, ECRS generally has more severe lesions; Soler et al.^24^ also showed high recurrence rates after surgery and poor treatment effect. In this study, ECRS was defined by tissue eosinophilic granulocyte ≥10/HPF. There were no significant differences in comparing eosinophilic granulocyte counts in the polyp tissue between the SIgE‐positive and the SIgE‐negative groups, which suggested that the eosinophilic granulocytes might not increase when CRS_w_NP patients have allergic reactions. It suggests that the relationship between the general allergic reaction status and the degree of infiltration and activation of eosinophilic granulocyte in local tissues is uncertain.^7^, ^8^ It shows that the stimulation of sensitinogen to SIgE‐positive CRS_w_NP patients does not induce an inflammatory response of eosinophilic granulocyte in the nasal polyps, but there is an independent pathophysiological mechanism of inflammation maintenance.^25^ About 62% of patients with negative in vitro allergy tests combined with severe paranasal sinus disease; they have elevated eosinophilic granulocyte in peripheral blood, and more than 50% of ECRS patients have no allergy.^26^

In this study, patients in the SIgE‐positive group with ECRS had significant differences compared with the other groups in terms of the subjective score of VAS symptoms and the objective scores of Lund‐Mackay CT, Lund‐Kennedy physical signs, and SNOT‐22 score indicating that in CRS_w_NP patients, the combination of allergic reaction and ECRS could aggravate the subjective symptoms and the objective physical signs of CRS_w_NP, thus Reducing the quality of life of patients with of CRS_w_NP. Kowalik et al.^27^ showed that preoperative and postoperative nasal endoscopic scores, paranasal sinus CT scores, and the incidence of asthma were associated with the tissue eosinophilic granulocyte. These results emphasize the important role of eosinophilic granulocyte in the pathogenesis of CRS_._ ^28^

For the treatment of CRSwNP, at present, it mainly depends on nasal endoscopic surgery and local or systemic drug treatment, such as glucocorticoids and macrolides; CRSwNP is associated with Th2 type inflammation and immune hypersensitivity comorbidities^29^ (such as asthma, atopic dermatitis and chronic urticaria). Glucocorticoids have a good effect on type 2 reaction and ECRSwNP, while macrolide antibiotics are applicable to non type 2 or non eosinophil infiltration type. we can divide the endoscopic sinus surgery for ECRSwNP into the following types: contour surgery (Nasalisation) to open each sinus as much as possible but retain the mucosa, contour surgery to remove ethmoid sinus mucosa, and contour surgery to remove all sinus mucosa (Reboot).^30^ However, even after adequate and standardized drug and surgical treatment, a considerable number of patients will still relapse.^18^, ^31^ Related studies at home and abroad have shown that eosinophilic inflammation and type 2 inflammation are important risk factors for recurrence of CRS patients.^32^, ^33^ CRSwNP patients characterized by eosinophilic inflammation and type 2 inflammation have high levels of immunoglobulin (Ig) E, interleukin (IL‐5), IL‐4 and IL‐13 in local tissues, often accompanied by eosinophilia in peripheral blood.^31^, ^34^, ^35^ Recently, biological agents targeting type 2 inflammation have been continuously developed for refractory CRSwNP patients with poor effects of conventional anti‐inflammatory and surgical treatment, and anti IgE monoclonal antibodies—Omalizumab and anti IL‐4 receptor α (IL‐4 receptor α), monoclonal antibodies Dupilumab, anti IL‐5 monoclonal antibodies Mepolizumab and anti IL‐5 receptor α (IL‐5 receptor α). Phase III clinical research data of monoclonal antibody Benralizumab have been published successively.^36^, ^37^, ^38^, ^39^, ^40^ Omalizumab, Dupilumab, and Mepolizumab have been approved by the United States or the European Union for clinical use in the treatment of patients with severe refractory CRSwNP. Although the above monoclonal antibody treatment of CRSwNP has not been approved in China, it can be used for refractory CRSwNP patients with asthma or atopic dermatitis. It is believed that in the near future, the clinical comprehensive treatment of CRSwNP will step into the era of biological therapy.

This study is a retrospective study and has some limitations. First, the sample size was relatively small. Second, various prediction methods have advantages and disadvantages, and there is a lack of mature prediction methods for CRS_w_NP. The relationship between local tissue IgE and eosinophilic granulocyte has not been established. Third, the impact mechanism of allergy on CRS needs to be further explored.

In conclusion, the VAS score, endoscopic score, and CT score might be higher in CRSwNP patients with SIgE‐positive combined ECRS.

## AUTHOR CONTRIBUTIONS

*Conception and design*: Jingmin Hu; *data curation*: Jingmin Hu; *formal analysis and visualization*: Le Wang; *investigation*: Le Wang; *writing, original draft*: Jingmin Hu and Ruixia Ma; All authors read and approved the final manuscript.

## CONFLICT OF INTEREST STATEMENT

The authors declare no conflicts of interest.

## ETHICS STATEMENT

This study protocol was reviewed and approved by the First People's Hospital of Yinchuan City (approval number: 20190608). The requirement for individual consent was waived by the committee because of the retrospective nature of the study.

## Data Availability

All data generated or analyzed during this study are included in this article. Further enquiries can be directed to the corresponding author.
